# Influence of TRPV1 on diabetes-induced alterations in thermal pain sensitivity

**DOI:** 10.1186/1744-8069-4-9

**Published:** 2008-03-01

**Authors:** Reddy M Pabbidi, Shuang-Quan Yu, Siying Peng, Romesh Khardori, Mary E Pauza, Louis S Premkumar

**Affiliations:** 1 Department of Pharmacology, Southern Illinois University School of Medicine, Springfield, IL 62702, USA; 2 Department of Medical Microbiology, Immunology and Cell Biology, Southern Illinois University School of Medicine Springfield, IL 62702, USA; 3 Department of Internal Medicine/Endocrinology, Metabolism and Molecular Medicine, Southern Illinois University School of Medicine Springfield, IL 62702, USA

## Abstract

A common complication associated with diabetes is painful or painless diabetic peripheral neuropathy (DPN). The mechanisms and determinants responsible for these peripheral neuropathies are poorly understood. Using both streptozotocin (STZ)-induced and transgene-mediated murine models of type 1 diabetes (T1D), we demonstrate that Transient Receptor Potential Vanilloid 1 (TRPV1) expression varies with the neuropathic phenotype. We have found that both STZ- and transgene-mediated T1D are associated with two distinct phases of thermal pain sensitivity that parallel changes in TRPV1 as determined by paw withdrawal latency (PWL). An early phase of hyperalgesia and a late phase of hypoalgesia are evident. TRPV1-mediated whole cell currents are larger and smaller in dorsal root ganglion (DRG) neurons collected from hyperalgesic and hypoalgesic mice. Resiniferatoxin (RTX) binding, a measure of TRPV1 expression is increased and decreased in DRG and paw skin of hyperalgesic and hypoalgesic mice, respectively. Immunohistochemical labeling of spinal cord lamina I and II, dorsal root ganglion (DRG), and paw skin from hyperalgesic and hypoalgesic mice reveal increased and decreased TRPV1 expression, respectively. A role for TRPV1 in thermal DPN is further suggested by the failure of STZ treatment to influence thermal nociception in TRPV1 deficient mice. These findings demonstrate that altered TRPV1 expression and function contribute to diabetes-induced changes in thermal perception.

## Background

Diabetic peripheral neuropathy (DPN) is a common chronic complication of diabetes mellitus [1]. Symptoms of this debilitating condition include progressive loss of thermal and tactile pain sensation [2,3]. Although, most individuals with diabetic neuropathy experience reduced perception, a fraction (~10%) experience painful symptoms [2]. Results from the Diabetes Control and Complications Trial [4] show that intensive glycemic control for 5 years reduces the incidence of neuropathy by 60% in individuals with T1D, suggesting that dysregulated glucose metabolism contributes to neuropathy. However, the fact that strict glucose regulation does not completely prevent DPN suggests additional mechanisms of pathogenesis exist [3,4]. Furthermore, sensory neuropathy can be detected in some individuals who have impaired glucose tolerance but may not meet the criterion for a diagnosis of frank diabetes [5]. The occurrence of hyperalgesia prior to hyperglycemia in some rodent disease models also suggests that aspects of altered sensory perception may be independent of glucose metabolism [6]. Additional studies suggest that insulin deficiency or insulin insensitivity could contribute to development of DPN [7,8]. Direct insulin signaling achieved by intrathecal administration of insulin reverses neuropathy in STZ-induced diabetes [9].

Transient receptor potential (TRP) ion channels are involved in sensing physical and chemical stimuli [10,11]. TRPV1 is a Ca^2+ ^permeant non-selective cation channel expressed predominantly by unmyelinated C-fibers and thinly myelinated Aδ fibers and plays a major role in inflammatory thermal sensation [12]. TRPV1 is activated by heat (~>43°C), protons, N-arachidonyl dopamine (NADA), anandamide and lipoxygenase metabolites of arachidonic acid [12-16]. Sensitization of TRPV1 by phosphorylation is mediated by numerous factors including, prostaglandins [17,18], bradykinin [19-21], glutamate [22], serotonin [23], histamine [24], ATP [25-27], trypsin [28,29] and nerve growth factor (NGF) [14,30]. Since TRPV1 is involved in inflammatory thermal sensation, it is logical to determine the expression and function of TRPV1 in DPN. Furthermore, capsaicin, a TRPV1 agonist has been shown to improve sensory perception in humans with DPN [31,32].

In this study, we have used STZ-induced and transgene-mediated diabetes models to study the role of TRPV1 in DPN. We show in these animal models of diabetes, DPN manifests as an initial phase of thermal hyperalgesia and a late phase of thermal hypoalgesia. These phenotypes are accompanied by up and down regulation of TRPV1, respectively. We further confirm our findings by the lack of these phenotypes in STZ-injected diabetic TRPV1 knock-out mice.

## Methods

### Induction of diabetes

All procedures used in this study were approved by the animal care and use committee at Southern Illinois University, School of Medicine and conformed according to NIH and institutional guidelines. All efforts were made to minimize the number of animals used and their suffering. The mice were housed in specific pathogen free barrier animal facility. Rodent laboratory chow (Laboratory Diet 5001, Nutrition International, Inc., Brentwood, MO, USA) and drinking water were provided *ad libitum*. Ins-HA.D2, B6.129S4-*Trpv1*^*tm*1*Jul*^/J (TRPV1 knock-out) and C57Bl6/J (background strain of TRPV1 knock-out) mice were housed in standard cages and maintained on a 12-h light/dark cycle at an ambient temperature of 22 ± 1°C. Mice were at least 6–10 weeks of age and weighed between 18 – 23 gms at the time of the experiments.

#### STZ-induced diabetes model

Age matched Ins-HA.D2 single transgenic mice, TRPV1 knock-out mice, and the back ground strain (C57Bl6/J) was used for STZ-induced diabetes. Diabetes was induced in mice by a single intraperitoneal injection of 200 mg/kg STZ (Sigma, St Louis), prepared fresh in saline adjusted to pH 4.5 with 0.1 N citrate buffer as described previously [33,34]. Control mice received citrate buffered saline alone. Although Ins-HA.D2 mice are genetically altered, HA expression does not affect glucose metabolism or neuronal function as measured by peripheral blood glucose levels and hot plate tests.

#### Double transgenic diabetes model (TCR-SFE/Ins-HA)

Mice expressing the TCR-SFE transgene were kindly provided by Dr. von Boehmer [35]. T cells from TCR-SFE mice express a T cell receptor (TCR) specific for the influenza hemagglutinin (HA) peptide 110–119 (SFERFEIFPK). Ins-HA.D2 (Insulin-hemaglutinin) transgenic mice express HA in islet beta cells and were kindly provided by Dr. Lo [36,37]. Both TCR-SFE and Ins-HA mice were maintained on the B10.D2 strain background. When TCR-SFE and Ins-HA mice are crossed, the resulting double transgenic (TCR-SFE/Ins-HA) mice become diabetic within approximately 4–6 weeks of birth.

#### TRPV1 deficient mice

The previously described B6.129S4-*Trpv1*^*tm*1*Jul*^/J (TRPV1 knock-out) mice and its background strain (C57Bl6/J) were purchased from the Jackson Laboratory (Bar Harbor, ME, USA) [38,39]. TRPV1KO mice were maintained as homozygous mutants in concordance with previous experiments [38,39].

### Blood glucose measurement

Glucose levels were quantitated with a One Touch Ultra blood glucose monitoring system (Life Scan, California) using whole blood obtained from the tail. Diabetes was defined as blood glucose concentrations greater than 299 mg/dl (16.7 mM) [40].

### Hot plate testing

All the mice used in this study were housed in the barrier facility. Mice were tested in the same room of barrier facility on the days the cages were not cleaned. Mice were placed individually on a Hot Plate Analgesia Meter (Harvard Apparatus, Boston, MA) maintained at a constant temperature of 52 ± 0.3°C after observing them for 5 five minutes in the cage. The paw withdrawal latency (PWL) is recorded as the time taken for the animal to exhibit a distinct pain behavior either by a hind paw lick or a characteristic hind paw flick (whichever occurs first). Mice that did not respond within 20 seconds were removed from the hot plate to prevent tissue damage. We did not find a significant difference in PWL either with habituation (1–2 hrs) or without habituation (5 min) inside the barrier facility. Hot plate testing was performed on randomly chosen animals from diabetic or control groups. After completing the test, the ear tags were read to place them in the appropriate groups. There was no significant effect of sex, therefore males and females were grouped together for further analyses. The PWL of control animals remained constant, any deviation that is significantly different from these values was considered as hyper (PWL <8 s) or hypoalgesic (PWL >12 s). The PWL was significantly higher (PWL >16 s) in TRPV1 knockout mice as compared to control mice.

### DRG neuronal cultures

After determining the PWL, the DRG were dissected from diabetic and control mice euthanized by deep anesthesia with isoflurane followed by decapitation. Isolated DRG were collected in HBSS (calcium- and magnesium-free) on ice and then enzymatically digested for 45 minutes at 37°C in 0.1% collagenase D, Worthington type 2 (Roche diagnostics, Indianapolis, IN) and 0.1% trypsin, type 1 (Sigma-Aldrich, St. Louis, MO). DRG were dissociated by trituration using fire-polished Pasteur pipettes. Cells were plated on 12 mm poly D-lysine coated glass coverslips placed in 24 well plates and grown in neurobasal medium (Gibco BRL, Grand Island, NY) supplemented with L-glutamine (2 mM, Invitrogen, Grand Island, NY) and 10 μl/ml B27-supplement (Gibco, Invitrogen corporation, GrandIsland, N.Y). Neurons were maintained at 37°C in a humidified atmosphere of 5% CO_2 _and were used within 8–10 hours.

### Whole cell patch clamp recording

For whole-cell patch-clamp recordings of cultured DRG neurons, the bath solution contained (in mM): 140 Na gluconate, 2.5 KCl, 10 HEPES, 1 MgCl_2_, and 1.5 EGTA. The pipette solution contained (in mM): 130 Na gluconate, 10 NaCl, 2.5 KCl, 10 HEPES, 1 MgCl_2_, and 1.5 EGTA. Both bath and pipette solutions were adjusted to pH 7.35 with NaOH. Ca^2+^-free solutions were used to avoid desensitization and tachyphylaxis of capsaicin-induced currents. Currents were recorded using a WPC 100 patch-clamp amplifier (E.S.F. Electronic, Goettingan, Germany). The capacitance and the series resistance were compensated. Data were digitized (VR-10B; Instrutech, Great Neck, NY) and stored on video tape. For analysis, data were filtered at 2.5 kHz (-3 dB frequency with an eight-pole low-pass Bessel filter; LPF-8; Warner Instruments, Hamden, CT) and digitized at 5 kHz. Current amplitudes were measured using Channel 2 (software kindly provided by Michael Smith, Australian National University, Canberra, Australia). The traces and graphs were made using Origin (Microcal Software, Northampton, MA).

### [^3^H]-Resiniferatoxin (RTX) binding

Hind paw skin and DRG were collected from non-diabetic and diabetic mice and placed immediately in ice-cold PBS containing 5 mM EDTA. Samples were lysed in binding buffer (in mM: 5 KCl, 5.8 NaCl, 0.75 CaCl_2_, 2 MgCl_2_, 137 sucrose, and 10 HEPES, pH 7.4) using a tissue homogenizer. Homogenates were centrifuged for 10 minutes at 1000 × *g *(4°C). Supernatants were collected and centrifuged again at 35,000 × *g *for 30 min (4°C) to obtain partially purified membrane fractions (pellets). Pellets were resuspended in 0.5 ml binding buffer and protein levels were quantitated using Biorad protein assay (Bio-Rad laboratories, Inc., CA).

Binding assay mixtures were set up on ice in glass tubes (Kimble Glass Inc.) and consisted of 100 μl binding buffer, 50 μl [^3^H] RTX (37 Ci/mmol specific activity, Perkin-Elmer Life Sciences) and 100 μl membrane preparation (~50 μg/assay tube). In each set of experiments, nonspecific binding was defined in the presence of 100 μl of cold 100 μM capsaicin (final concentration). Reaction mixtures were incubated in a 37°C shaking (50 rpm) water bath for 1 hour. Reactions were terminated by chilling assay mixtures on ice for 5 minutes. To reduce non-specific binding, 100 μl of bovine α_1_-acid glycoprotein (2 mg/ml; Sigma) was added into the binding mix and incubated for an additional 10 minutes. After incubation, samples were filtered over Whatman GF/B glass fiber filters (Brandel, Gaithersburg, MD) pretreated with 0.05% polyethyleneimine. The radioactive content of each filter was determined using a Beckman Instruments (Fullerton, CA) liquid scintillation counter.

### Immunohistochemistry

After determining the PWL, mice were anesthetized by using isoflurane and L5 segments of spinal cord, DRG and ventral paw skin were collected in the 4 % paraformaldehyde following transcardial perfusion of control and diabetic mice. Tissues were fixed for 3 hours in fixative and incubated in 15% and 30% sucrose for 12 hours. Samples were quickly frozen in liquid nitrogen. Dorsal horn and paw tissue were cut into 20 μm thick sections and DRG were cut 10 μm thick sections. The paw skin was longitudinally cut into 20 μm sections. Sections were incubated with polyclonal rabbit anti-TRPV1 antibody (Calbiochem, PC 420, 1:50) and monoclonal mouse anti-NeuN antibody (Chemicon, MAB 377, 1:100) for 2 hours at room temperature, then incubated with Rhodamine Red (TM)-X donkey anti-rabbit IgG (Jackson 711-295-152, 1: 100) and FITC donkey anti-mouse IgG (Jackson, 715-095-151, 1: 100) for 1 hour at room temperature, then spread on slides, mounted with mounting medium (Biomed Gel/Mount) and covered by coverslips. Images were taken by using confocal microscope (Olympus Fluoview, Melville, NY) keeping a constant exposure time between tissue sections under 10 times magnification. Immunohistochemistry was performed in a fashion that the phenotype was not known to the experimenter.

### Immunohistochemistry analysis

The animals that clearly exhibited hyperalgesic (PWL< 7s) and hypoalgesic (PWL >12 s) phenotypes were used for immunohistochemical studies. Only the full size sections (which included the soma-dense lateral parts and the fiber-dense central part) were taken into account for quantification. Sections were randomly selected from 3–4 mice of each phenotype, whose numbers and stain intensity (total gray values) of the TRPV1-expressing neurons were counted by Image J. While analyzing the sections of the paw skin, only the TRPV1 staining in the small region between the second foot pad and the fifth foot pad was taken into account. 8–9 sections in this region from 4 mice of each phenotype were selected randomly, whose total gray values were measured by ImageJ. The mean gray value was calculated by the Image J software, and product of the area and the mean gray value of the selected region were regarded as the total gray value of the staining region. The gray value from the background (an area where no staining is observed) is obtained and subtracted. We have used confocal microscope and kept the background intensity constant while analyzing the data. Moreover, we have subtracted the background value to get an objective measure.

### Data analysis

For behavioral experiments, mixed model analysis was performed using SAS software, which includes both fixed events (age and time after diabetes onset) and random events (number of subjects) with repeated measures ANOVA. The comparisons were made between control group and diabetic groups. Data are shown as mean ± S.E.M. (standard Error of Mean) Data are considered significant at p < 0.05.

For all other experiments, data are shown as mean ± S.E.M. Significance is tested using unpaired *Student's t*-test and the data are considered significant at p < 0.05.

All the chemicals used in this study were obtained from Sigma (St. Louis, MO).

## Results

### Changes in blood glucose level, body weight and thermal pain sensitivity in mice injected with STZ

**Figure 1:**
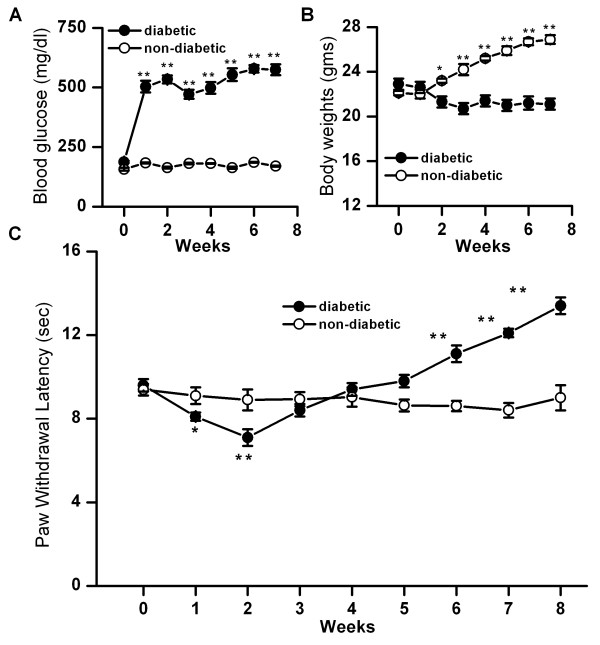
Changes in blood glucose level, body weight and thermal pain sensitivity in mice (Ins-HAD2) injected with STZ. A. After intraperitoneal injection of STZ, within the first week, blood glucose levels significantly increased (filled circles, n = 11, p < 0.001) and remained constant throughout the course of the experiment as compared to control non-diabetic mice (Ins-HAD2) (open circles, n = 6). B. The body weight of diabetic mice remained constant (filled circles, n = 11), but the body weight of control mice increased steadily (open circles, n = 6, p < 0.001) C. PWL of diabetic mice determined every week after injection of STZ show significant changes: exhibited a phase of hyperalgesia (filled circles; n = 33, p < 0.001) between weeks 1 and 4; a phase of hypoalgesia (filled circles; n = 8, p < 0.001) as compared to control non-diabetic mice (open circles, n = 19). Note that the number of mice is decreasing over the course of experiments because unhealthy mice were not tested further on the hot plate. Asterisks (*, **) represent p < 0.05 and p < 0.001, respectively.

STZ is toxic to pancreatic beta cells and is commonly used to induce type 1 diabetes in rodents for the study of DPN because disease induction is both rapid and reliable. In this study, 6–10 weeks old mice of either sex were used and observed for a period of 7–9 weeks after the onset of diabetes. Ins-HA.D2 single transgenic mice were injected with a single dose of STZ (200 mg/kg). Blood glucose levels were significantly elevated within one week after treatment and remained elevated for the entire course of the study (pre-injection, 157 ± 4.4; week 1 post-STZ injection, 503.5 ± 24.6; week 6 post-STZ, 578.6 ± 15.6 mg/dl, n = 11, p < 0.001) as compared to control non-diabetic mice (pre-injection, 197 ± 6.1; week 1 vehicle control, 183.6 ± 2.2; week 6 vehicle control, 186 ± 1.6 mg/dl, n = 6) (Fig. 1A). As disease progressed, body weights of STZ-treated animals remained stagnant, whereas the body weights of control animals increased steadily (control week 1, 22.1 ± 0.2; control week 6, 26.7 ± 0.3 gms, n = 6; post-STZ week 1, 22.9 ± 0.5; post-STZ week 6, 21.2 ± 0.6, gms n = 11, p < 0.001) (Fig. 1B). As indicated in the Method Section, mice were tested for thermal pain sensitivity by measuring PWL using a hot plate maintained at 52 ± 0.3°C. An early phase of thermal hyperalgesia occurred 1–3 weeks post-STZ treatment, which paralleled the onset of hyperglycemia (control week 2, 8.9 ± 0.5 s, n = 19; diabetic week 2, 7.1 ± 0.4, n = 33, p < 0.001) (Fig. 1C). Hyperalgesia was followed by a second phase of apparently normal PWL (control week 4, 9 ± 0.5 s, n = 19; diabetic week 4, 9.4 ± 0.3 s, n = 18) and then a final phase of hypoalgesia beginning at week 6 (control week 8, 9 ± 0.8 s, n = 19, diabetic week 8, 13.4 ± 0.7 s, n = 8, p < 0.001) (Fig. 1C). As the disease progressed, the mice which did not appear healthy were not used further, hence the 'n' values decreased over the study period. Together these results indicate that STZ-induced diabetic animals exhibited an initial phase of hyperalgesia and a phase of hypoalgesia.

### Changes in blood glucose level, body weight and thermal pain sensitivity in double transgenic mice model of diabetes

**Figure 2:**
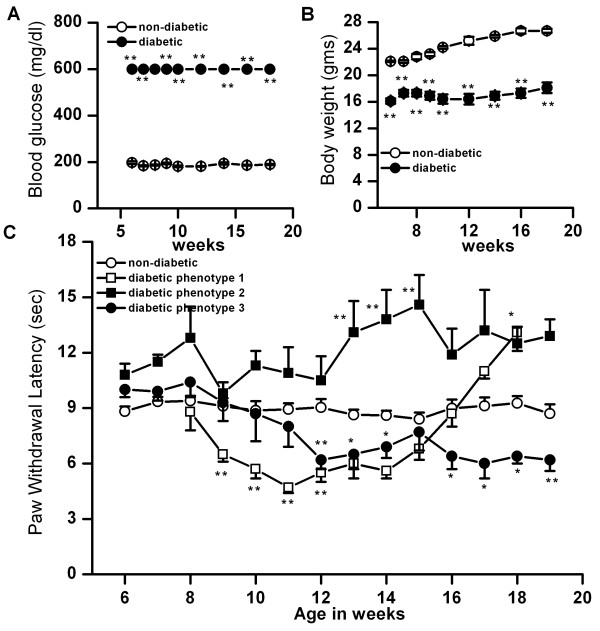
Changes in blood glucose level, body weight and thermal pain sensitivity in a double transgenic mice (TCR-SFE/Ins-HAD2) model of diabetes. A. By six weeks of age, double transgenic mice became diabetic (blood glucose levels >600 mg/dl) and remained high (filled circles, n = 9, p < 0.001) throughout the course of the experiment as compared to non-diabetic control (Ins-HAD2) mice (open circles, n = 6). B. Body weights of TCR-SFE/Ins-HAD2 mice remained constant (filled circles, n = 9), while control non-diabetic mice (Ins-HAD2) gained weight steadily (open circles, n = 6, p < 0.001) C. PWL measured from the age of 6 weeks to 18–19 weeks revealed three phenotypes as compared to control non-diabetic mice (Ins-HAD2) (open circles, n = 19). The first group exhibited an initial phase of hyperalgesia (open squares, n = 12, p < 0.001) followed by a phase of hypoalgesia (n = 8, p < 0.05). The second group showed no hyperalgesic phase, but became hypoalgesic (filled squares, n = 8, p < 0.001). The third group became hyperalgesic and remained hyperalgesic throughout the course of the study (filled circles, n = 12. Asterisks (*, **) represent p < 0.05 and p < 0.001, respectively.

To study DPN in a model system devoid of exogenous toxins or infectious agents that might themselves influence nerve function, we examined sensory perception in a cohort of diabetic double transgenic TCR-SFE/Ins-HA mice. These mice become diabetic within 4 to 6 weeks of age due to CD4 T cell mediated destruction of pancreatic beta cells [36,37]. Autoimmune diabetes occurs spontaneously and exhibits complete penetrance with a predictable rapid disease course. Diabetes was well established among TCR-SFE/Ins-HA double transgenic mice at 6 weeks of age; average blood glucose levels were typically at the maximum limit of detection (>600 mg/dl) (Fig. 2A). Age-matched single transgenic mice (TCR-SFE or Ins-HA.D2) served as controls and remained euglycemic over the course of study. Average body weights were also significantly lower among TCR-SFE/Ins-HA mice as compared to single transgenic control mice (week 6 single transgenic, 22.1 ± 0.2; week 18, 26.7 ± 0.3 gms, n = 6; week 6 double transgenic, 16.1 ± 0.4 gms; week 18, 18.1 ± 0.3 gms, n = 9, p < 0.01)(Fig. 2B).

Thermal sensitivity was determined from 6 weeks to 17–19 weeks of their age using a hot plate as described in the Methods Section. As seen with STZ-treated mice, deviation from normal PWL was characterized by a phase of hyperalgesia and a phase of hypoalgesia. However, unlike STZ-induced diabetic mice, a uniform change in PWL was not observed in double transgenic diabetic mice. To best explain the observations, the animals were separated into three groups based on the following criteria. First, the animals were divided into two groups based on the animals that developed hypoalgesia vs. the animals remained hyperalgesic. The group of animals that showed hyperalgesia was further subdivided because a subset of animals exhibited only hyperalgesic phenotype.

One group of diabetic SFE/Ins-HA mice was characterized by an initial stage of hyperalgesia (week 11, 4.7 ± 0.3 s, n = 12, as compared to control single transgenic, 8.9 ± 0.3 s, p < 0.01, n = 19) followed by an intermediate phase of apparently normal pain sensitivity (week 16, 8.7 ± 0.7 s, n = 12, as compared to controls 9 ± 0.5 s, n = 19) and a late phase of thermal hypoalgesia, which lasted for the remainder of the study (week 18, 13.1 ± 1 s n = 8, as compared to controls, 9.3 ± 0.4 s, n = 19, p < 0.05) (Fig. 2C). The second group of diabetic SFE/Ins-HA mice exhibited hypoalgesia without an early phase of hyperalgesia between weeks 13 and 16 (week 14, 14.6 ± 1.6 s, n = 8 as compared to controls, 8.6 ± 0.3, n= 19, p < 0.01) (Fig. 2C). Finally, the third group of diabetic SFE/Ins-HA mice showed a significant decrease in PWL (week 8, 10.4 ± 0.8 s; week 14, 6.9 ± 0.6 s; week 18, 6.4 ± 0.4 s, n = 12, p < 0.05) as compared to controls (week 8, 9.4 ± 0.3 s, n = 19; week 14, 8.6 ± 0.3 s, n = 19, week 18, 9.3 ± 0.4 s, n = 19) (Fig. 2C). Thus, hyper and hypoalgesic phenotypes were observed in diabetic TCR-SFE/Ins-HA mice, but the phases were not clearly demarked as in STZ treated animals.

### Changes in blood glucose level, body weight and thermal pain sensitivity in TRPV1 knock-out mice injected with STZ

**Figure 3:**
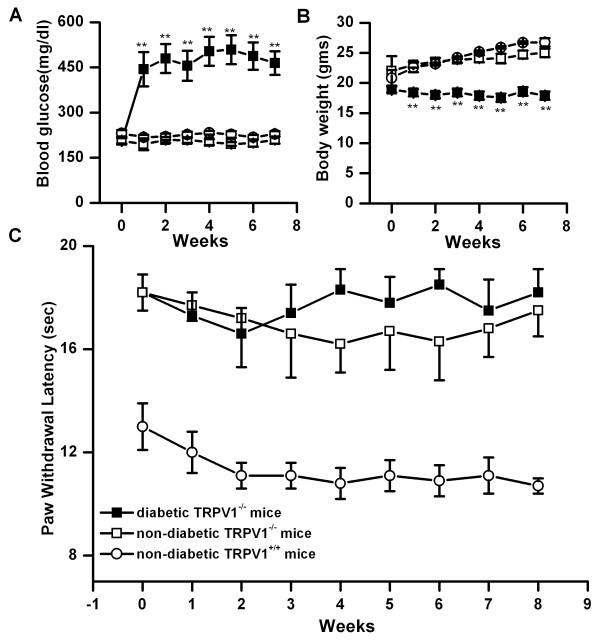
Changes in blood glucose level, body weight and thermal pain sensitivity in TRPV1 knock-out mice (B6.129S4-*Trpv1*^*tm*1*Jul*^/J) injected with STZ. A. After intraperitoneal injection of STZ, within the first week, blood glucose level significantly increased (filled squares, n = 14, p < 0.001) and remained constant throughout the course of the experiment as compared to non-diabetic TRPV1 knock-out mice (open squares, n = 6) or the background strain of TRPV1 knock-out mice (C57BL/6J) (open circles n = 15). B. The body weight of TRPV1 knock-out diabetic mice remained constant (filled squares, n = 14), but the body weight of non-diabetic knock-out mice increased steadily (open squares, n = 6, p < 0.001). C. PWL of TRPV1 knock-out diabetic mice, determined every week did not show any significant change (filled squares, n = 14) as compared to control non-diabetic TRPV1 knock-out mice (open squares, n = 6) and the background strain of TRPV1 knock-out mice (open circles n = 15). Note that PWL is significantly higher in TRPV1 knock-out mice as compared to the background strain. Asterisk (**) represents p < 0.001.

To determine whether TRPV1 plays a role in the altered thermal pain sensitivity observed in diabetic mice, we treated TRPV1^-/- ^mice with 200 mg/kg STZ or vehicle and then determined PWL. TRPV1^-/- ^mice became hyperglycemic following STZ treatment, (preinjection 206 ± 11; week 7 post-STZ, 464 ± 39 mg/dl; n = 14, p < 0.001) compared with vehicle injected control non-diabetic TRPV1^-/- ^mice (preinjection, 206 ± 9; week 7 post-vehicle, 208.7 ± 11 mg/dl, n = 6) (Fig. 3A). STZ-treated diabetic TRPV1^-/- ^mice did not gain weight (prediabetic, 20.1 ± 0.5; diabetic week 7, 17.9 ± 0.7, n = 14), whereas the body weight of vehicle injected non-diabetic TRPV1^-/- ^mice increased steadily (preinjection 21.1 ± 1.3; week 7 post-vehicle, 26.7 ± 0.4, n = 6, p < 0.05 from the preinjection). Thermal pain sensitivity of these mice was tested upto 8 weeks after the onset of diabetes. PWL of STZ treated diabetic TRPV1^-/- ^mice did not differ from vehicle injected control non-diabetic TRPV1^-/- ^mice (STZ treated TRPV1^-/- ^mice week 4, 18.3 ± 0.8, n = 14; week 8, 18.2 ± 0.9, n = 14; vehicle treated TRPV1^-/- ^mice week 4, 16.2 ± 1.1, n = 6; week 8, 17.5 ± 1 s, n = 6) (Fig. 3C). Together these data demonstrate that STZ-induced diabetes does not alter thermal pain sensitivity in TRPV1 knock-out mice further suggesting TRPV1 plays a major role in diabetes-induced altered thermal pain sensitivity. In our experimental conditons, in contrast to previous studies, we noticed a higher PWL in TRPV1 knock-out mice as compared to non-diabetic wild type mice (Ins-HA.D2 or C57BL/6J mice) suggesting that TRPV1 is necessary for acute thermal sensation [39]. We used Ins-HA.D2 mice as a control non-diabetic mice and parameters such as thermal pain sensitivity and blood glucose levels did not vary with background strain of TRPV1 knock-out mice that is C57BL/6J mice (Ins-HA.D2 mice, pretreatment 10 weeks of age, PWL 9.3 ± 0.6 s, n = 14, blood glucose levels 213.9 ± 9.8, n = 23; C57BL/6J mice pretreatment 10 weeks of age PWL 10.8 ± 0.4 s, n = 15, blood glucose levels 231.16 ± 5.6, n = 15).

### Alterations in TRPV1-mediated whole cell currents in DRG neurons obtained from diabetic mice

**Figure 4:**
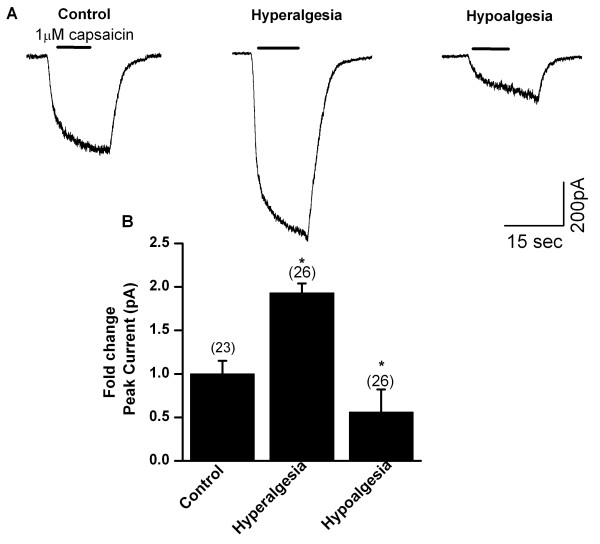
Altered capsaicin-evoked TRPV1-mediated whole cell currents in diabetic mice. Capsaicin (1 μM)-evoked TRPV1-currents were recorded from acutely dissociated DRG neurons from age matched diabetic and non-diabetic mice. **A**. Representative traces of capsaicin-evoked TRPV1 currents show that the amplitude is larger in hyperalgesic mice and smaller in hypoalgesic mice as compared to responses in control mice. **B**. Summary graph showing an average fold change in TRPV1-mediated peak currents is significantly larger in hyperalgesic (n = 26 cells, p < 0.05) and smaller in hypoalgesic mice (n = 26 cells, p < 0.05) as compared to age matched control mice (n = 23). Number in the parenthesis represents the number of cells and the asterisks (*) represents p < 0.05.

In order to determine if TRPV1 channel function changes as thermal perception increases or decreases in diabetic mice, we recorded capsaicin-induced whole cell currents by patch clamp techniques using acutely dissociated DRG neurons from hyperalgesic, hypoalgesic and normal mice from both age matched non-diabetic and STZ-induced diabetic mice and transgenic diabetic mice. Dissociated neurons were obtained after confirming the phenotype by PWL. Neurons were grown in the absence of trophic factors to avoid post translational and post transcriptional modification and used within 8 to 10 hrs. Whole-cell currents from these neurons were recorded using different concentrations of capsaicin. Peak current responses to 1 μM capsaicin were significantly larger (1056 ± 136 pA, n = 26 cells from 6 mice) (1.91 ± 0.12 fold, p < 0.05) from DRG obtained from hyperalgesic mice as compared to the age matched control mice (545 ± 83 pA, n = 23 cells from 6 mice). In DRG obtained from hypoalgesic mice, TRPV1-mediated currents were smaller (292 ± 75 pA, n = 26 cells from 6 mice) (0.56 ± 0.26 fold; p < 0.05) as compared to age matched control mice (545 ± 119 pA, n = 14 cells from 3 mice) (Fig. 4B). Similarly, hyperalgesic diabetic transgenic mice, the current amplitude was larger (906 ± 84 pA, n = 26 cells from 4 mice) as compared to age matched control mice (499 ± 49 pA, n = 23 cells from 4 mice). These results demonstrate that altered TRPV1 currents correlate with altered thermal pain sensitivities in diabetic mice. We have used near saturating concentrations of capsaicin and the differences in current amplitude suggest that the TRPV1 protein content as a result of altered transcriptional regulation is higher and lower in hyper and hypoalgesic mice, respectively rather than phosphorylation state of TRPV1, where they showed prolongation of decay phase [41].

### Determination of [^3^H]-RTX binding in diabetic mice

**Figure 5:**
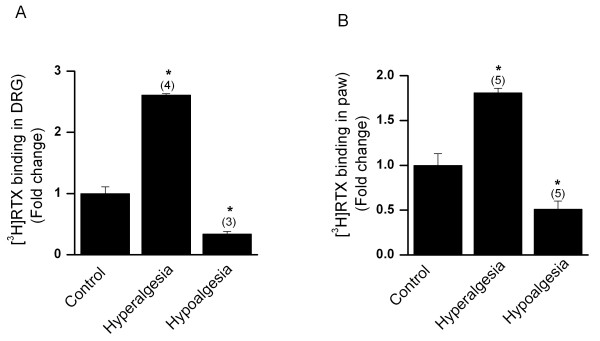
[^3^H]RTX binding in DRG neurons and paw skin obtained from STZ-induced diabetic mice. **A**. Diabetic hyperalgesic and hypoalgesic mice DRG exhibited higher (n = 4 experiments, p < 0.05) and lower (n = 3 experiments, p < 0.05) TRPV1 levels, respectively as compared to control non-diabetic mice. **B**. Paw tissue obtained from STZ-induced diabetic hyperalgesic and hypoalgesic mice expressed higher (n = 5 experiments, p < 0.05) and lower (n = 5 experiments, p < 0.05) TRPV1 levels, respectively as compared with control non-diabetic mice. Number in the parenthesis represents the number of experiments performed and the asterisk (*) represents *p *< 0.05.

Changes in capsaicin-induced currents may result from altered receptor sensitivity as well as expression. Therefore, to confirm that the TRPV1 protein content is different, TRPV1 levels were quantitated by using [^3^H]-RTX binding assays in DRG obtained from STZ treated diabetic or transgenic diabetic or vehicle injected non-diabetic mice (Fig. 5A) Compared to DRG obtained from vehicle treated non-diabetic control mice (167 ± 20 fmol/mg tissue), [^3^H]-RTX binding was significantly increased in DRG from hyperalgesic mice (2.6 ± 0.02 fold, n = 4, p < 0.05) and decreased in hypoalgesic mice (0.34 ± 0.04 fold, n = 3, p < 0.05). The DRG obtained from hypoalgesic transgenic diabetic mice showed lower (0.34 ± 0.09 fold, n = 4, p < 0.05) [^3^H]-RTX binding as compared to control mice).

Alterations in DRG TRPV1 expression may be secondary to changes that occur in the periphery. To determine the state of TRPV1 protein expression in the periphery, we quantitated TRPV1 levels in paw skin using [^3^H]-RTX binding assays (Fig. 5B). Similar to data obtained from DRG, [^3^H]-RTX binding was higher in paw skin from hyperalgesic mice (1.81 ± 0.05 fold, n = 5, p < 0.05) and lower in hypoalgesic mice (0.51 ± 0.09 fold, n = 5, p < 0.05) as compared to non-diabetic control mice (188.8 ± 26.5 fmol/mg tissue). The paw skin of hypoalgesic transgenic diabetic mice showed lower [^3^H]-RTX binding (0.29 ± 0.06 fold, n = 4, p < 0.05) as compared to paw skin harvested from control nondiabetic mice. Thus, changes in TRPV1 protein expression at both the peripheral nerve terminals and DRGs may contribute to the abnormal thermal sensitivity observed in diabetic mice. As the *K*_d _of this radio ligand in most tissues is ~0.1 nM, it is estimated that > 90% of the available receptors are labeled with this concentration [42,43]. Therefore, the increase in radioligand binding observed in the diabetic mice with [^3^H]-RTX reflects an increase in the total TRPV1 levels.

### Determination of TRPV1 expression using immunohistochemistry

**Figure 6:**
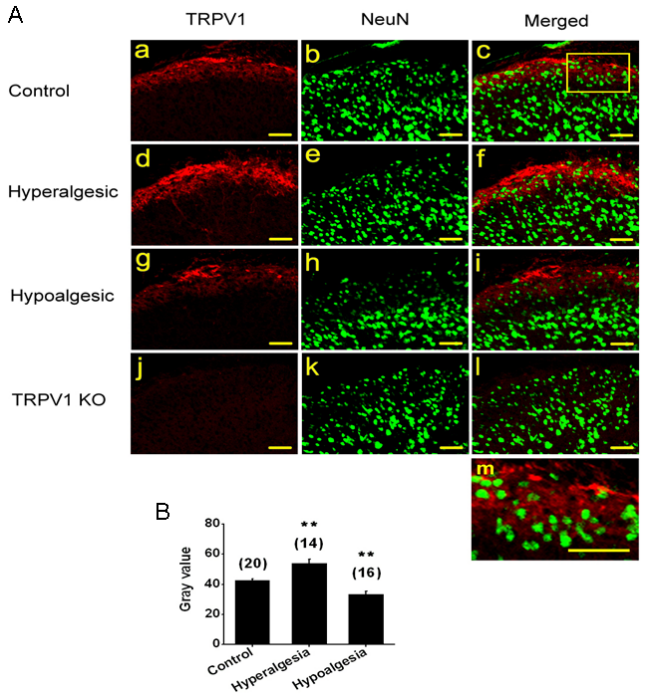
Altered TRPV1 staining in spinal cord dorsal horn of diabetic mice. **A**. Representative pictures of TRPV1 and NeuN staining from a control (a, b, c), STZ-induced diabetic hyperalgesic (d, e, f)), hypoalgesic (g, h, i), and non-diabetic TRPV1 knock-out mice (j, k, l). An enlarged segment of Af is shown in Am. **B**. Mean gray values of TRPV1 staining in dorsal horn was significantly increased (n = 14 sections from 3 mice, p < 0.01) in hyperalgesic mice and significantly decreased (n = 16 sections from 3 mice, p < 0.01) in hypoalgesic mice as compared to age matched control non-diabetic mice. Number in the parenthesis represents the number of TRPV1 stained sections and the asterisks (**) represents p < 0.01 obtained from student unpaired t-test. Scale bar is 50 μm.

To confirm [^3^H]-RTX binding results and further examine TRPV1 expression, immunohistochemistry was performed on spinal cord, DRG and paw skin tissue harvested from STZ treated or transgenic diabetic hyperalgesic, hypoalgesic mice and age matched non-diabetic mice (Ins-HA.D2). TRPV1 is expressed in the central terminals of DRG neurons, which synapse with the second order dorsal horn neurons at laminae I and II of the spinal cord (Fig. 6). Expression of TRPV1 was significantly higher in the DH from hyperalgesic diabetic mice (54 ± 2.4 gray value, n = 14 sections from 3 mice, p < 0.01) and lower in hypoalgesic mice (33 ± 2, n = 16 sections from 3 mice, p < 0.01) as compared to age matched non-diabetic mice (42.5 ± 1, n = 20 sections from 3 mice) (Fig. 6B). Similarly, in transgenic hyperalgesic and hypoalgesic diabetic mice the TRPV1 expression in DH was higher (71 ± 4 gray value, n = 6 sections from 3 mice, p < 0.01) and lower (41 ± 1.5 gray value, n = 8 sections from 3 mice, p < 0.05), respectively as compared to control animals (52 ± 3 gray value, n = 8 sections from 3 mice, p < 0.05). In order to confirm the specificity of anti-TRPV1 antibody binding, we used TRPV1 deficient mice, which exhibited no staining for TRPV1. The general morphology and the distribution of neurons in diabetic mice were normal as indicated by the staining of the neuronal marker, NeuN (Fig. 6A b, e, h, k).

**Figure 7:**
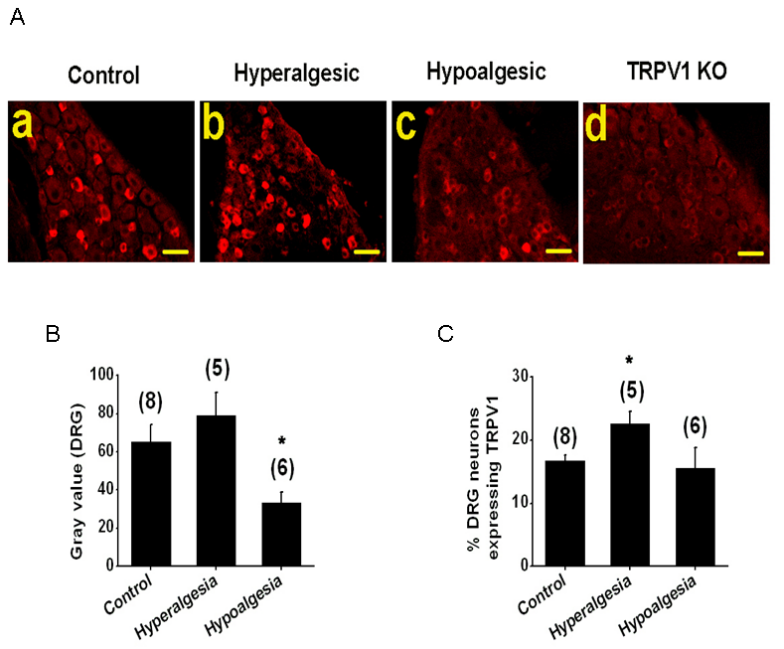
Altered TRPV1 staining is observed in DRG obtained from STZ-induced diabetic mice. **A**. Representative TRPV1 immunofluorescent pictures of DRG sections from control non-diabetic (a), STZ-induced diabetic hyperalgesic (b), hypoalgesic mice (c) and non-diabetic TRPV1 knock-out mice (d). **B**. Summary graph showing increase in mean gray values of TRPV1 staining (n = 5 sections from 4 mice, p < 0.058) in hyperalgesic DRG and decrease in TRPV1 gray value in hypoalgesic diabetic mice (n = 6 sections from 4 mice, p < 0.05) as compared to control non-diabetic mice. **C**. Percentage of TRPV1-expressing neurons in total DRG neuron was higher (n = 5 sections from 4 mice, p < 0.05) in hyperalgesia and did not change in hypoalgesic (n = 6 sections) diabetic mice as compared to age matched control non-diabetic mice. Note that TRPV1knock-out mice sections did not exhibit any TRPV1 staining serving as negative control. Number in the parenthesis represents the number of TRPV1 stained sections and the asterisk (*) represents p < 0.05. Scale bar is 50 μm.

Immunohistochemistry performed on DRG from hyperalgesic, hypoalgesic or non-diabetic mice (Fig. 7) revealed that TRPV1 protein expression was more extensive among DRG obtained from hyperalgesic as compared to non-diabetic mice (Fig. 7A) and was reduced among tissues from hypoalgesic compared to non-diabetic mice. To better quantitate anti-TRPV1 staining, gray scale analyses were performed. Overall fluorescence was highest in DRG tissue sections from hyperalgesic mice (78.8 ± 12.01, n = 5 sections from 4 mice), followed by control tissues (65.3 ± 9.37, n = 8 sections from 4 mice) and then tissues from hypoalgesic mice (33.3 ± 5.6, n = 6 sections from 4 mice, p < 0.05) (Fig. 7B).

Recent studies suggest that TRPV1 expression may occur ectopically on large diameter DRG neurons in diabetic mice [44]. In contrast, other studies suggest that long standing diabetes results in neuronal loss [45]. To determine if changes in TRPV1 staining among DRG might be due to either ectopic expression or neuronal loss, we determined the average percentage of neurons expressing TRPV1 per section (Fig. 7C). The percentage of TRPV1 positive neurons was similar for tissues from hypoalgesic (15.6% ± 3.2, n = 6 sections from 4 mice) and control (16.7% ± 0.9, n = 8 sections from 4 mice, p < 0.71) mice. However, more neurons expressed TRPV1 in DRG from hyperalgesic (22.6% ± 1.9, n = 5 sections from 4 mice p < 0.05) mice. Collectively, these data suggest that the thermal hypoalgesia observed secondary to protracted dysglycemia in STZ-treated mice in our study is not due to loss of TRPV1 positive DRG neurons, however ectopic expression of TRPV1 on large diameter neurons cannot be excluded as a possible explanation for the increased TRPV1 expression observed among DRG isolated from hyperalgesic diabetic mice.

**Figure 8:**
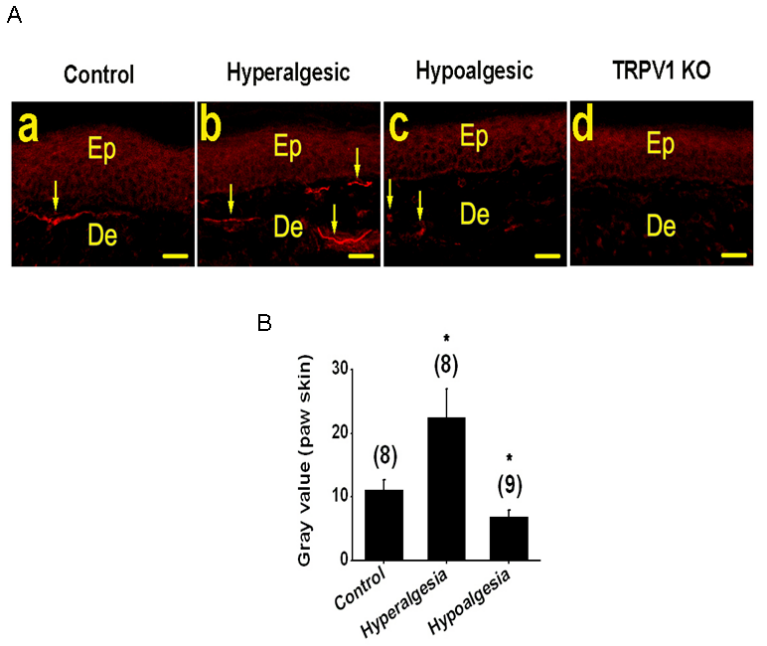
Altered TRPV1 staining in paw skin tissue obtained from STZ-induced diabetic mice. **A**. Representative TRPV1 immunofluorescent pictures of paw skin sections from control non-diabetic (a), STZ-induced diabetic hyperalgesic (b), hypoalgesic mice (c) and non-diabetic TRPV1 knock-out mice (d). Arrows indicate the TRPV1-expressing fibers in dermis (De). Background staining was consistently observed in the epidermis (Ep) of all sections including TRPV1 knock-out mice. **B**. Summary graph showing increased (n = 8 sections from 4 mice, p < 0.05) and decreased (n = 9 sections, p < 0.05) mean gray values of TRPV1 staining in diabetic hyperalgesic and hypoalgesic fibers of the paw dermis layer as compared with control non-diabetic mice (n = 8 sections from 4 mice). Number in the parenthesis represents the number of TRPV1 stained sections and the asterisk (*) represents p < 0.05. Scale bar is 50 μm.

Expression of TRPV1 is elevated in the fibers of the dermis of skin from hyperalgesic diabetic mice and decreased in skin from hypoalgesic mice compared to control non-diabetic mice (Fig. 8A). Overall fluorescence was higher in the dermal regions of skin from hyperalgesic mice (22.5 ± 4.5, n= 8 sections from 4 mice) compared to control mice (11.1 ± 1.6, n = 8 sections from 4 mice, p < 0.05) (Fig. 8B). In contrast, overall fluorescence was reduced in the dermis of skin from hypoalgesic mice (6.7 ± 1, n = 9 sections from 4 mice, p < 0.05) compared to control non-diabetic mice. TRPV1 immunoreactivity was absent from the dermis of paw skin sections obtained from TRPV1 deficient mice (Fig. 8Ad). Thus, altered TRPV1 levels at the central terminals of sensory neurons in the spinal cord, DRG neurons and the peripheral terminals indicate that altered expression of TRPV1 is responsible for the observed differences in thermal sensitivity.

## Discussion

### Altered thermal sensation in mouse models of diabetes

In diabetes, peripheral neuropathy (DPN) can manifest as focal, autonomic, or peripheral sensory neuropathy [46]. Altered thermal perception can occur as a result of sensory neuropathy [2]. In this study, we used both STZ- and transgene- (TCR-SFE/Ins-HA; [47]) mediated Type 1 diabetes (T1D) models to determine whether TRPV1 expression and function were altered with changes in thermal pain sensitivity in diabetes. STZ was used because of its frequent use in similar studies of DPN, rapid and efficient rate of hyperglycemia induction, and ability to produce and sustain a 'moderate degree' of hyperglycemia (~450–500 mg/dl). However, STZ has toxic effects on cells other than islet beta cells that might confound results making it important to use other models of diabetes. Unlike diabetes induced by STZ, TCR-SFE/Ins-HA double transgenic mice become hyperglycemic spontaneously by 4–6 weeks of age. Disease parallels human diabetes in that it is immune-mediated, affects both genders equally and results from selective destruction of islet beta cells.

STZ-induced diabetic mice exhibited two phases of altered thermal pain sensitivity, an initial transient phase of hyperalgesia followed by a phase of hypoalgesia. Similarly, when subjected to the hot plate test, transgenic diabetic mice exhibited two phases of altered thermal pain sensitivity. However, the transition to different phases was not uniform in these animals. Careful examination of thermal sensitivities of individual mice over the course of the study allowed for classification of three unique phenotypes. One group exhibited thermal sensitivities similar to those observed with STZ treated mice. Thus, phenotype 1 was characterized by a period of hyperalgesia followed by a sustained phase of hypoalgesia. A second group of diabetic TCR-SFE/Ins-HA mice exhibited thermal hypoalgesia at approximately the same age as observed for phenotype 1 mice but lacked an early hyperalgesic phase (phenotype 2). The third group of animals became slightly hyperalgesic and remained hyperalgesic during the course of the study (phenotype 3). It is unclear why these unique thermal sensitivity phenotypes are observed among syngenic mice with uniformly dysregulated glucose control. Together these behavioral studies show altered heat sensation, a finding in diabetic neuropathy, is present in our animal models [48,49].

### Evidence of TRPV1 involvement in the altered thermal nociception of diabetic mice

We tested the hypothesis that the altered pain sensation is due to changes in TRPV1 because the altered temperature sensitivity corresponds to the temperature range that activates TRPV1. We provide four lines of evidence to support the involvement of TRPV1. First, thermal sensitivity was not altered among STZ-induced diabetic TRPV1 deficient mice demonstrating the thermal sensitivity in diabetic mice is dependent on TRPV1. However, a significantly higher PWL was observed in TRPV1 knock-out mice as compared to wild type mice with similar back ground strain suggesting that TRPV1 knock-out mice has deficiency in acute thermal pain sensation (Fig. 3C).

Second, TRPV1 mediated capsaicin induced currents in DRG neurons collected from mice exhibiting hyperalgesia or hypoalgesia were significantly larger or smaller, respectively, compared to currents obtained from euglycemic control mice. The change could be as a result of enhanced expression or sensitivity. The increase was observed in saturating concentrations of capsaicin confirming increased TRPV1 protein levels rather than as a result of increased sensitivity due to phosphorylation of TRPV1 [41].

Third, RTX binding assays demonstrated that TRPV1 expression parallels thermal sensitivity. DRG neurons from hyperalgesic mice bound more RTX and hypoalgesic mice bound less RTX than DRGs from age-matched euglycemic control mice. It is conceivable that peripheral TRPV1 expression might influence expression in DRG; therefore, we determined TRPV1 levels from the paw skin. Peripheral TRPV1 expression mirrored that observed in DRG neurons. Thus, changes in TPRV1 expression parallel the thermal sensitivity observed among diabetic mice and are seen both at peripheral tissue and in DRG.

Fourth, we used immunohistochemical localization of TRPV1 in DH of the spinal cord, DRG neurons and the paw skin to confirm changes in TRPV1 expression detected in RTX binding assays and to determine if increased RTX binding was the result of ectopic TRPV1 expression (on neurons other than C and Aδ fibers). In the DH, there was no obvious co-localization of TRPV1 with the neuronal marker NeuN, suggesting that the distribution of TRPV1 in DH is predominantly in fibers projecting from DRG rather than in the cell body of DH neurons. We observed a significant increase in expression of TRPV1 in DH, DRG and paw skin from hyperalgesic diabetic mice as compared to age-matched control mice. Significant decreases were also observed among tissues harvested from hypoalgesic diabetic mice. Furthermore, the number of neurons expressing TRPV1 is altered in tissues harvested from hypoalgesic mice, but the total number of neurons remains unchanged. This is important to address because it has been suggested that there may be neuronal loss in diabetes leading to altered thermal sensitivity [45]. We found the number of neurons expressing TRPV1 was higher in hyperalgesic mice, consistent with a previous report [44]. There was no change in the number of neurons expressing TRPV1 in hypoalgesic mice relative to controls.

### Possible down stream events leading to altered TRPV1 expression

In diabetes, the link between abnormal thermal sensitivity and hyperglycemia resulting from insulin deficiency is not clear. Here, we have shown that TRPV1 is a major player, but questions still exist with respect to how TRPV1 function and expression are modulated in diabetes. Previously, we showed that insulin and IGF-1, acting via the IGF-1 receptor, potentiates TRPV1 current by activating PKC [50]. Based on this observation, the long lasting hypoalgesic phenotype could be explained by insulin deficiency leading to downregulation of TRPV1 expression and function. However, it is counterintuitive that lack of insulin results in hyperalgesic phenotype, which can not be explained readily. It is possible that an abrupt reduction in insulin levels as occurs in STZ-treated mice could trigger compensatory enhancement of TRPV1 expression resulting in hyperalgesia. On the other hand, diabetes in our transgenic mouse model results from a slower, more sustained destruction of beta cells that may lead to a different course of neuropathic progression. Based on this premise, hyperalgesia in some patients (~10%) may be explained by rapid metabolic decompensation, perhaps a sudden onset. An increase in TRPV1 expression in DRGs occurs as a transient compensatory mechanism to decreased TRPV1-mediated neuronal activity similar to what happens during deafferentation. Furthermore, hyperglycemia is known to induce oxidative stress, as a result a slight increase in ROS is known to activate transcriptional machinery enhancing the expression of TRPV1, at the same time higher levels of ROS can lead to neuronal cell death [45,51,52]. Another important finding we have made in our recent studies is that some mice injected with STZ were not hyperglycemic but were hyperalgesic. These results have led to a new hypothesis that STZ has a direct effect and may contribute to hyperalgesia independent of hyperglycemia. This may be one of the reasons that the hyperalgesic phenotype is clearly seen in STZ induced diabetes [53].

We have recently showed Increased sensitivity of TRPV1 in STZ-induced diabetic mice has been shown that increased thermal sensitivity results from sensitization of TRPV1, possibly through PKC [33,41,54]. In chronic pain conditions, increased TRPV1 expression occurs via MAPK activity [10,55]. Furthermore, increased enzyme activity from hyperglycemia, specifically PKCβ is implicated in several diabetic complications including thermal hyperalgesia [56,57]. All these studies offer an explanation for the hyperalgesic phenotype, which is rarely observed in diabetes, or goes undetected because of its transient nature. The significant finding of this study is that the predominant phenotype observed over time is thermal hypoalgesia as a result of reduced TRPV1 expression and function. If untreated, this might lead to more serious complications, which include changes in microvascular function due to decreased release of CGRP, neuronal loss, development of gangrene and total loss of sensation leading to amputation. Careful monitoring of hypoalgesic phenotype and a prompt treatment with insulin may decrease the progression and the severity of the disease.

### Consequences of enhanced and reduced TRPV1 expression and function other than abnormal thermal sensation

TRPV1 is present in areas that are not exposed to sufficient changes in temperature to cause channel activation and is widely believed to have functions other than temperature sensation. TRPV1 is detected throughout the neuroaxis by RT-PCR [58] and identification of specific ligands such as NADA and anandamide in certain brain regions confirms a role in the CNS [15]. The nature of the receptors involved in this response and their roles in the CNS is not clearly understood, but suggest TRPV1 may have direct and/or indirect effects on neurotransmitter release [15,59-61]. TRPV1 expressing neurons in pancreas are also shown to play a role in beta cell function and diabetes pathoetiology [62]. TRPV1 is also expressed in the bronchi, urinary bladder, heart and blood vessels [63-66]. Activation of TRPV1 in sensory nerve endings supplying heart and blood vessels causes release of multiple vasoactive agents [67]. Therefore, as diabetes progresses, modulation of TRPV1 has the potential to contribute to many complications such as CNS, cardiovascular, respiratory and urinary disturbances.
